# Links between Insulin Resistance and Periodontal Bacteria: Insights on Molecular Players and Therapeutic Potential of Polyphenols

**DOI:** 10.3390/biom12030378

**Published:** 2022-02-28

**Authors:** Katy Thouvenot, Teva Turpin, Janice Taïlé, Karine Clément, Olivier Meilhac, Marie-Paule Gonthier

**Affiliations:** 1 Université de La Réunion, Inserm, UMR 1188 Diabète Athérothrombose Thérapies Réunion Océan Indien (DéTROI), 97490 Saint-Denis de La Réunion, France; katy.thouvenot@gmail.com (K.T.); turpin.teva@gmail.com (T.T.); janice.taile@univ-reunion.fr (J.T.); olivier.meilhac@inserm.fr (O.M.); 2 Nutrition and Obesity, Systemic Approaches (NutriOmics), INSERM, Sorbonne Université, 75013 Paris, France

**Keywords:** insulin resistance, diabetes, obesity, periodontal bacteria, inflammation, oxidative stress, polyphenols

## Abstract

Type 2 diabetes is a metabolic disease mainly associated with insulin resistance during obesity and constitutes a major public health problem worldwide. A strong link has been established between type 2 diabetes and periodontitis, an infectious dental disease characterized by chronic inflammation and destruction of the tooth-supporting tissue or periodontium. However, the molecular mechanisms linking periodontal bacteria and insulin resistance remain poorly elucidated. This study aims to summarize the mechanisms possibly involved based on in vivo and in vitro studies and targets them for innovative therapies. Indeed, during periodontitis, inflammatory lesions of the periodontal tissue may allow periodontal bacteria to disseminate into the bloodstream and reach tissues, including adipose tissue and skeletal muscles that store glucose in response to insulin. Locally, periodontal bacteria and their components, such as lipopolysaccharides and gingipains, may deregulate inflammatory pathways, altering the production of pro-inflammatory cytokines/chemokines. Moreover, periodontal bacteria may promote ROS overproduction via downregulation of the enzymatic antioxidant defense system, leading to oxidative stress. Crosstalk between players of inflammation and oxidative stress contributes to disruption of the insulin signaling pathway and promotes insulin resistance. In parallel, periodontal bacteria alter glucose and lipid metabolism in the liver and deregulate insulin production by pancreatic β-cells, contributing to hyperglycemia. Interestingly, therapeutic management of periodontitis reduces systemic inflammation markers and ameliorates insulin sensitivity in type 2 diabetic patients. Of note, plant polyphenols exert anti-inflammatory and antioxidant activities as well as insulin-sensitizing and anti-bacterial actions. Thus, polyphenol-based therapies are of high interest for helping to counteract the deleterious effects of periodontal bacteria and improve insulin resistance.

## 1. Introduction

Diabetes is a metabolic disease characterized by chronic hyperglycemia and constitutes a major global health problem since its progression can lead to multiple complications impacting the blood vessels, heart, nerves, kidneys and eyes. According to the International Diabetes Federation, 537 million adults currently suffer from diabetes [1]. Type 2 diabetes accounts for 90% of diabetes and results from impaired glucose homeostasis and insulin resistance. Insulin resistance can be associated with multiple etiologic factors and refers to a complex pathological condition in which insulin-dependent cells have an inappropriate cellular response to insulin. Overweight and obesity are the strongest risk factors of insulin resistance and type 2 diabetes [2].

A bidirectional relationship has been established between type 2 diabetes and oral infections such as periodontitis. Periodontitis is a periodontal disease characterized by chronic inflammation of the tooth-supporting tissue called the periodontium, leading to its destruction [3] and designated as the sixth complication of diabetes. It is well known that diabetes is associated with an increased risk of periodontitis [4,5,6]. However, growing literature data and clinical studies report that the reverse relationship is also true: people with severe periodontitis have a higher probability of developing type 2 diabetes [7,8,9]. Furthermore, a recent meta-analysis of cohort studies also confirmed this bidirectional association [10]. This is supported by several in vivo studies demonstrating that exposition to periodontal bacteria is associated with the development of insulin resistance and glucose intolerance in mice [11,12,13]. Interestingly, therapeutic management of periodontitis improves metabolic parameters of type 2 diabetic patients [14,15].

Although there is increasing evidence of an association between periodontal bacteria and insulin resistance, the molecular mechanisms involved are still unknown [16]. One of the most mentioned hypotheses involves the translocation of periodontal bacteria components into the bloodstream through inflammatory lesions of the periodontium [17]. These bacterial components might contribute to systemic inflammation and reach distant tissues, including adipose tissue, skeletal muscle and the liver [18]. Locally, molecular events involving inflammatory and redox alterations may impair the insulin signaling pathway, leading to insulin resistance. Given the causal roles of oxidative stress and inflammation in several pathologies, including obesity and diabetes, there is a high interest in the biological activities of plant polyphenols able to exert antioxidant and anti-inflammatory effects. Moreover, polyphenols may exhibit insulin-sensitizing and anti-bacterial properties that may counteract the deleterious effects of periodontal bacteria and improve insulin resistance [19].

The aim of this study is to review the current knowledge about the possible molecular mechanisms linking insulin resistance and periodontal bacteria. Firstly, the molecular events responsible for the insulin signaling pathway and glucose uptake are presented. Secondly, the parameters characterizing periodontal infections and their impact on the disruption of insulin response in target tissues comprising adipose tissue, skeletal muscle, and the liver, as well as insulin production by pancreatic β-cells, are described. Furthermore, plant polyphenol-based strategies to reduce inflammatory and redox alterations caused by periodontal bacteria during insulin resistance are discussed as possible innovative pharmacological approaches.

## 2. Molecular Events Related to Insulin Signaling Pathway and Insulin Resistance

### 2.1. Insulin

Insulin is an anabolic peptide hormone consisting of 51 amino acids organized in two chains: an α chain and a β chain linked by two disulphide bridges [20]. This hormone is produced by the pancreatic β-cells of the Langerhans islets and is essential for the regulation of blood glucose levels. Insulin mediates its effects by binding to specific receptors on the plasma membrane of target cells and activating intracellular signaling pathways leading to glucose uptake.

After a meal, the glucose contained in the food bolus is absorbed by enterocytes, translocated into the liver via the portal vein and released into the bloodstream. The pancreas, which detects an increase in blood glucose levels, secretes insulin, which acts on various tissues and organs to induce glucose uptake and maintain blood glucose levels at a physiological concentration close to 1 g/L. The main targets of insulin are the liver, skeletal muscle and adipose tissue [21]. Insulin also acts on the pancreas to inhibit the secretion of glucagon by the pancreatic α-cells.

**Figure 1:**
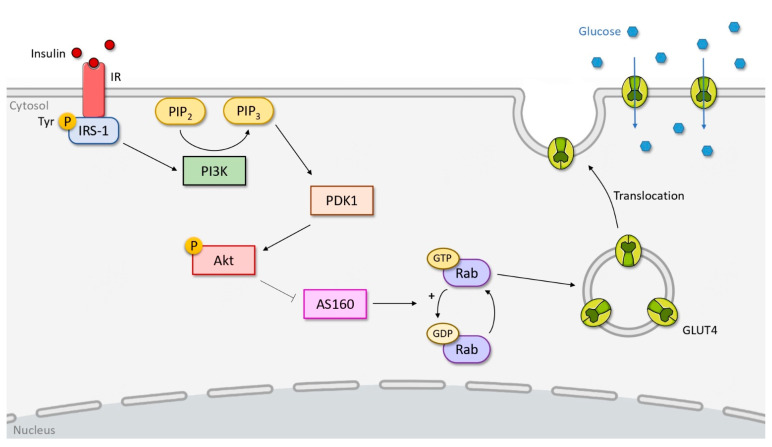
Insulin signaling pathway mediating glucose uptake. Insulin binds to its receptor (IR) and activates its tyrosine kinase activity. IR catalyzes the phosphorylation of tyrosine residue of IRS-1 protein, which in turn activates PI3K. Subsequently, PI3K metabolizes PIP2 to PIP3, leading to PDK1 activation. PDK1 activates Akt by phosphorylation on threonine residue. Activated Akt inhibits AS160 protein. Under basal condition, AS160 hydrolyses Rab-GTP to its inactive form Rab-GDP via its Rab GAP domain. Inhibition of AS160 by Akt promotes the translocation of GLUT-4 transport vesicles to the cell plasma membrane, allowing glucose uptake.

Skeletal muscle cells and adipose cells are called insulin-dependent cells. In these cells, glucose uptake is ensured by glucose transporter type 4 (GLUT-4), whose presence on the cellular plasma membrane is dependent on insulin (Figure 1). In the absence of insulin, GLUT-4 is retained in the cytoplasm in storage vesicles [22]. The binding of insulin to its receptor induces a signaling cascade involving the phosphoinositide 3-kinase (PI3K)/serine/threonine protein kinase (Akt) pathway and leads to the exocytosis of these vesicles and the translocation of GLUT-4 transporter at the cellular plasma membrane, allowing the entry and storage of glucose in the cell.

In the liver, unlike in skeletal muscle cells and adipocytes, glucose uptake is carried out by the GLUT-2 transporter, which is non-insulin-dependent. Thus, glucose uptake by hepatocytes is not dependent on or directly influenced by insulin. In these cells, insulin stimulates glycogen synthesis from glucose through activation of glycogen synthase and inhibition of gluconeogenesis, leading to a reduction of blood glucose levels.

### 2.2. Insulin-Stimulated Glucose Uptake Signaling Pathway

The insulin receptor (IR) is a tetrameric complex belonging to the tyrosine kinase receptor superfamily, composed of two extracellular α-subunits that bind insulin and two transmembrane β-subunits carrying tyrosine kinase activity, linked together by disulphide bonds.

Upon arrival at the target cell surface, insulin binds to the α-subunits of the IR and induces conformational changes in the receptor, leading to the autophosphorylation of the tyrosine residues of the β-subunits (Figure 1). Then, the IR activates a substrate from the insulin receptor substrate (IRS) protein family called IRS-1 through the phosphorylation of specific tyrosine residues. These phosphorylated residues form docking sites for proteins with an Src homology 2 (SH2) or protein tyrosine-binding (PTB) domain [23]. Activated IRS-1 binds to PI3K via its p85 subunit through its SH2 domain, leading to the activation of its p110 catalytic domain. This event allows PI3K to catalyze the conversion by the phosphorylation of phosphatidylinositol-4,5-bisphosphate (PIP2) to phosphatidylinositol-3,4,5-triphosphate (PIP3), which recruits both the phosphoinositide-dependent kinase-1 (PDK1) and Akt, also known as protein kinase B (PKB), to the cellular plasma membrane. Subsequently, PDK1 activates Akt by the phosphorylation of threonine residue, which phosphorylates the protein Akt-substrate of 160 kDa (AS160).

AS160 carries a Rab GTPase activating protein (GAP) domain, which, under basal condition, hydrolyses Rab-bound GTP to its inactive Rab-bound GDP form, resulting in the retention of the glucose transporter GLUT-4 in the cytoplasm [24]. Phosphorylation of AS160 by Akt leads to the inactivation of its Rab GTPase activity and increased formation of Rab-GTP, which promotes translocation and subsequent fusion of GLUT-4 transport vesicles to the plasma membrane for glucose uptake (Figure 1).

### 2.3. Insulin Resistance

Insulin resistance refers to a condition in which insulin-sensitive cells have a subnormal response to physiological concentrations of insulin. This occurs when the insulin signaling pathway engaged downstream of the IR is impaired. A defect of the insulin response impairs glucose uptake by adipose tissue and skeletal muscle and reduces glycogen synthesis by the liver, leading to the establishment of a hyperglycemic state.

As a result of this insulin resistance, the pancreas first enters into a “compensatory period” in which β-cells hypertrophy and produce more insulin [25]. However, this hypersecretion of insulin cannot be maintained and will lead to a failure of the secretory function of β-cells and insulinopenia. Chronic hyperglycemia due to insulin resistance will also lead to glucotoxicity, inducing the loss of β-cells through apoptosis.

From an etiologic point of view, insulin resistance has been mainly related to high-fat and high-carbohydrate diets leading to fat mass gain in overweight and obese patients. Nevertheless, the mechanism explaining how excessive adiposity promotes insulin resistance, pancreatic β-cell dysfunction, hyperinsulinemia and hyperglycemia still needs to be investigated due to conflicting evidence [26]. On the one hand, some authors proposed that increased insulin resistance and insulinemia in obese patients are related to the ability of pancreatic β-cells to sense the need to secrete more insulin, in parallel with the capacity of tissues such as the liver, kidney and skeletal muscle to sense the need to clear less insulin to maintain normoglycemia [27,28]. On the other hand, other authors reported that insulin resistance is higher in people with obesity than in people who are lean, even when both groups are matched by basal glycemia and hepatic and muscle insulin sensitivity [29]. This raises the possibility that excessive fat mass per se causes unique alterations in the crosstalk between glycemia and insulin resistance independent of insulin sensitivity. Accordingly, it was demonstrated that increased adiposity could induce insulin secretion in obese patients, and the associated deterioration of β-cell function is a determinant of impaired fasting glucose leading to type 2 diabetes [30].

Interestingly, clinical trials reporting that insulin resistance and body weight are closely correlated also show that weight-loss diet interventions time-dependently lead to a decrease in fat mass, insulin resistance and major associated disorders such as blood pressure or β-cell dysfunction [31,32]. The study from Bondonno et al. [33] conducted on 54,787 participants investigated the association between diabetes and the consumption of diets rich in flavonoid micronutrients from the polyphenol family. It was found that participants with the highest total flavonoid intake had a 19% lower risk of diabetes, in part through a reduction of body fat. Likewise, the association of a lifestyle intervention with an energy-restricted Mediterranean diet and exercise program was reported to promote weight loss, improve glycemic control and insulin sensitivity, and reduce the inflammatory status and lipid profile markers related to cardiovascular risk factors [34]. Insulin resistance and hyperglycemia control in obese patients were also reported to be improved by bariatric surgery known to promote significant fat mass loss [35,36]. A recent study comparing the glycemic control in patients with obesity and type 2 diabetes who underwent bariatric surgery (BS) to those receiving medical treatment (MT) indicated a diabetes remission rate at 1 year, reaching 59% in the BS group and 0.4% in the MT group [37].

Overall, from a technical point of view, clinical biomarkers mostly explored to investigate insulin resistance in clinical trials include the measurement of fasting glycemia and insulinemia, oral glucose tolerance and circulating glycated hemoglobin A1c (HbA1c), and the calculation of parameters such as HOMA-IR index reflecting insulin resistance or HOMA-B index reflecting β-cell function [30,31,32,37].

### 2.4. Mechanisms of Insulin Signaling Disruption

**Figure 2:**
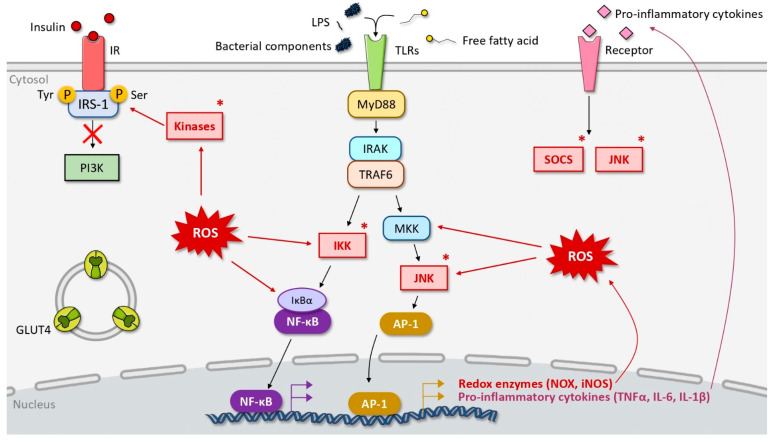
Molecular mechanisms impairing insulin signaling. Periodontal bacteria components and free fatty acids bind to TLRs at the plasma membrane of the target cell. TLRs recruit MyD88 and induce the activation of IRAK and TRAF6 proteins. This leads to the induction of NF-κB and MAPK pathways involving the transcriptional factors NF-κB and AP-1, respectively. These pathways promote the secretion of pro-inflammatory cytokines such as TNF-α, IL-6 and IL-1β. At the cell plasma membrane, cytokines bind to specific receptors and induce the activation of JNK and SOCS3. IKK and JNK are involved in signaling pathways downstream of TLRs, and SOCS3 induces insulin resistance by serine phosphorylation of IRS-1. Inactivation of IRS-1 impairs insulin signaling, resulting in the retention of GLUT-4 in the cytoplasm. In parallel, NF-κB/AP-1 pathway activates the expression of genes encoding ROS-producing enzymes like NOX and iNOS. Excessive intracellular ROS levels contribute to insulin resistance by activating IKK and JNK, which directly inhibit IRS-1, and by inducing NF-κB and MAPK pathways that lead to the production of pro-inflammatory cytokines.

Insulin resistance results from the impairment of the signaling cascade downstream of the insulin receptor (IR). Different mechanisms might contribute to the dysregulation of the insulin signaling pathway, but many insulin resistance inducers activate IRS kinases targeting the IRS-1 protein (Figure 2). Indeed, IRS-1 has many potential sites of serine phosphorylation that downregulate its activity. Many of these kinases are activated along insulin-unrelated pathways, as in the case of the inhibitor of nuclear factor kappa B kinase subunit beta (IKKβ), c-Jun NH_2_-terminal kinase (JNK) [38] and AMP-activated protein kinase (AMPK) [39]. It was reported that the mutation of IRS-1 serine phosphorylation sites protects mice against fat-induced insulin resistance [40]. Other mechanisms of insulin resistance include the reversion of the insulin-induced tyrosine phosphorylation of IRS-1, IRS degradation and decreased GLUT-4 expression [41]. Even though insulin resistance can be associated with multiple molecular mechanisms and has not been fully elucidated, two major phenomena are clearly related to insulin resistance, namely inflammation and oxidative stress.

#### 2.4.1. Inflammation

Inflammation has always been associated with insulin resistance. Numerous inflammatory cytokines and mediators, including tumor necrosis factor-α (TNF-α), interleukin-6 (IL-6) and monocyte chemoattractant protein 1 (MCP-1), are upregulated during insulin resistance [42]. Moreover, the inhibition of these mediators is associated with improving insulin sensitivity [43]. This impact of inflammatory mediators on insulin sensitivity can be mediated by various molecular mechanisms.

Two major signaling pathways are activated during inflammation and linked to insulin resistance: the nuclear factor-kappa B (NF-κB) pathway and the JNK/AP-1 pathway (Figure 2). Importantly, the activation of these pathways also mediates the activation of IKKβ and JNK1 serine kinases in the NF-κB and JNK/AP-1 pathways, respectively, and these kinases phosphorylate IRS-1 on serine, leading to its inactivation [38]. Interestingly, the absence of both JNK1 and IKKβ pathways results in improved insulin sensitivity and enhanced insulin receptor signaling in mouse models of obesity [44,45]. These pathways can be activated by numerous stimuli and lead, in turn, to inflammatory mediators involved in insulin resistance. Among inflammatory mediators, TNF-α and interleukin-1β (IL-1β) have been reported to mediate insulin resistance by IKKβ and JNK1-induced IRS-1 serine phosphorylation [46,47].

These signaling pathways are also involved downstream of Toll-like receptors (TLRs) and mediate the production of pro-inflammatory cytokines and chemokines like TNF-α, IL-6, IL-1β and MCP-1 (Figure 2) [48]. TLRs and TLR4, in particular, are known to be activated by free fatty acids (FFA) and the bacterial endotoxins called lipopolysaccharides (LPS). Subsequently, the NF-κB pathway is induced by the TLR-mediated signaling pathway and mediates a pro-inflammatory response. Indeed, after activation of TLR, the adaptor MyD88 binds to TLR and induces a signaling pathway involving interleukin-1 receptor-associated kinase (IRAK)-1 and 4, and TNF receptor-associated factor 6 (TRAF6), leading to the activation of the transcriptional factors NF-κB and AP-1 by phosphorylation of the IKK complex and JNK, respectively (Figure 2). Of note, these pro-inflammatory mechanisms have already been associated with periodontal bacteria LPS [49].

Inflammatory mediators are also associated with non-IRS-1 related insulin resistance. IL-6 induces the activation of the suppressor of cytokine signaling proteins SOCS1 and SOCS3 (Figure 2), which promote IRS ubiquitylation and degradation [50]. IL-1β downregulates IRS-1 gene expression [51]. In parallel, nitric oxide (NO) has been reported to reduce PI3K-Akt activity by s-nitrosylation of Akt [52].

Notably, the adipose tissue plays a key role in inflammation-induced insulin resistance as a massive source of inflammatory cytokines and specific adipokines, including leptin, resistin and adiponectin [53]. Both leptin and resistin are pro-inflammatory adipokines overproduced during obesity and associated with insulin resistance. Inversely, adipokine adiponectin is an anti-inflammatory adipokine that exerts pleiotropic anti-inflammatory, antioxidant and insulin-sensitizing effects. However, adiponectin production is downregulated during obesity and is inhibited by pro-inflammatory mediators such as IL-6 and TNF-α.

#### 2.4.2. Oxidative Stress

Oxidative stress is defined as an imbalance between the production of highly reactive species and antioxidant molecules. Reactive oxygen species (ROS) can be classified into free radicals that contain at least one unpaired valence electron, such as superoxide ion (•O_2_^−^) and hydroxyl (•OH), and a non-radical such as hydrogen peroxide (H_2_O_2_) [54]. The mitochondria are the main intracellular source of ROS as the mitochondrial respiratory chain produces ROS during cell respiration. ROS are also generated during enzymatic reactions involving NADPH oxidase (NOX), xanthine oxidase, cytochrome p450 and peroxidases [55]. These reactive species are continuously produced at low levels in the course of biological reactions and are essential for several physiological processes, including protein phosphorylation, cell signaling pathways, differentiation, activation of transcriptional factors, apoptosis, immunity and defense against infections by microorganisms [56]. In physiological situations, cells deploy antioxidant defenses to balance ROS production and keep it at low levels. This antioxidant defensive system is mainly based on antioxidant enzymes such as superoxide dismutase (SOD), catalase and glutathione peroxidase (GPx), whose production is mediated by the redox-sensitive transcriptional factor, nuclear factor erythroid 2-related factor 2 (Nrf2) [57]. Oxidative stress emerges when this antioxidant defense system is not enough to counterbalance the production of ROS.

Numerous cellular and metabolic dysfunctions can contribute to ROS overproduction and oxidative stress, including endoplasmic reticulum stress and increased advanced glycation end-product (AGE) formation. During obesity, excessive intracellular accumulation of fatty acid content in adipose cells leads to mitochondria dysfunction and increases ROS production. In parallel, the hyperglycemic condition leads to an AGE increase [58]. Of note, the activation of TLRs mediated by intestinal and periodontal bacterial stimuli also results in oxidative stress [49].

Oxidative stress exerts a deleterious impact on glycemic control by impairing glucose uptake. Cellular models of insulin resistance are characterized by elevated ROS levels, and treatment with antioxidant species improves insulin resistance [59]. Indeed, during oxidative stress, high intracellular ROS levels activate NF-κB, JNK and mitogen-activated protein kinase (MAPK) pathways [44] (Figure 2). As described above, the involvement of these pathways results in the activation of the serine kinases JNK1 and IKKβ, which impair insulin signaling pathways by serine-phosphorylation of IRS-1 [47]. Concomitantly, the activation of such pro-inflammatory signaling pathways enhances the secretion of pro-inflammatory cytokines such as TNF-α, IL-6 and IL-1β, which in turn contribute to altered insulin signaling (Figure 2). In parallel, the expression of genes coding for ROS-producing enzymes such as NOX and inducible nitric oxide synthase (iNOS) is induced by the pro-inflammatory NF-κB/AP-1 pathway (Figure 2). Excessive ROS react with other cellular components such as proteins, lipids and DNA, damaging molecular structures and cellular functions [55]. In particular, ROS can cause lipid peroxidation, leading to damaged cellular membranes and circulating lipoproteins. These reactive species can also damage proteins, inducing conformational modifications and loss or impairment of function/enzymatic activity, and damage DNA, possibly leading to mutagenesis. These damages contribute to cellular dysfunctions. Importantly, there is real crosstalk between inflammation and oxidative stress since excessive ROS levels promote the secretion of various pro-inflammatory cytokines that induce oxidative stress [59]. Both mechanisms maintain each other, and their synergistic action may contribute to insulin resistance onset and aggravation.

## 3. Molecular Players Linking Insulin Resistance and Periodontal Bacteria

### 3.1. Periodontitis and Associated Main Periodontal Bacteria

Periodontitis is a multifactorial oral disease characterized by chronic inflammation of the tissues supporting the tooth. The development of this condition results from a dysbiosis of the oral microbiota, which induces a deleterious inflammatory response by the host, leading to progressive destruction of the periodontal tissues and, ultimately, to the loss of teeth. Periodontitis is epidemiologically linked with several diseases such as cardiovascular diseases, obesity, diabetes, neurodegenerative pathologies and non-alcoholic fatty liver disease [60,61].

Oral dysbiosis is characterized by the development of anaerobic Gram-negative bacteria within the dental plaque in the depths of periodontal pockets, where oxygen levels are very low. Even if there is great inter-individual variability in the microbiota found within periodontal pockets, some bacteria are statistically abundant during periodontitis. This is the case for the three bacteria of Socranski’s red complex: *Porphyromonas gingivalis* (*P. gingivalis*), *Tannerella forsythia* and *Treponema denticola*. Other bacteria such as *Prevotella intermedia*, *Fusobacterium nucleatum* and *Campylobacter rectus* are also abundant during periodontitis. These periodontal bacteria have various virulence factors that can negatively impact the host and participate in the pathogenesis of periodontitis. Indeed, several constituents of the outer membrane of these gram-negative bacteria, such as fimbriae, LPS, flagellin and outer membrane vesicles (OMVs), can participate in and influence the inflammatory response of the host [62].

Among these constituents, particular attention is paid to LPS, recognized as major constituents of the wall of these bacteria and highly immunoreactive molecules. In humans, the action of LPS is mainly mediated by the TLRs family such as TLR4 and TLR2 [63], which are expressed, on the one hand, by immune cells and, on the other hand, by many cell types, including adipocytes [64], skeletal muscle cells [65], hepatocytes [66] and pancreatic β-cells [67]. Activation of these receptors induces NF-κB and MAPK pathways [63], leading to the secretion of pro-inflammatory cytokines, such as TNF-α and IL-6, and promoting oxidative stress [49], which is particularly demonstrated by LPS from *P. gingivalis*. Fimbriae play a key role during biofilm formation, bacterial attachment to host tissues and invasion into host cells. As a late colonizer of the subgingival biofilm, *P. gingivalis* fimbriae play an important role in its establishment by allowing it to interact and aggregate with earlier colonizers already present within the biofilm [68,69]. Fimbriae are also thought to play a crucial role in the invasion of oral epithelial cells by periodontal bacteria [70]. OMVs are small vesicles released into the extracellular environment by Gram-negative bacteria carrying multiple biomolecules, including adhesion molecules and virulence factors [71]. *P. gingivalis* OMVs have been reported to mediate the transport of proteases [18]. Among periodontal pathogens, *P. gingivalis* has the characteristic to produce specific proteases called gingipains, which highly contribute to making it a major periodontopathic pathogen. Gingipains are cysteine proteases highly conserved among *P. gingivalis* strains. There are two types of gingipains, comprising arginine-specific gingipains (Rgp) and lysine-specific gingipains (Kpg), that collectively account for 85% of the extracellular proteolytic activity of *P. gingivalis* [72]. These proteases are able to inactivate pro-inflammatory mediators and make the bacteria able to evade innate immunity [73]. Kadowaki et al. [74] have nicely demonstrated that using inhibitors of gingipains can suppress the pathogenicity of *P. gingivalis*.

Periodontal pathogens have been associated with multiple metabolic diseases, including cardiovascular diseases, liver diseases, dyslipidemia, obesity and diabetes [61]. The common hypothesis proposed involves the translocation of periodontal pathogens and related components into systemic circulation due to the breakdown of oral epithelium integrity. Indeed, severe periodontitis is associated with the destruction of the surrounding and supporting tissues of the teeth, including the gums, cementum, periodontal ligament and alveolar bone. This degradation results from chronic inflammation mediated by a complex immune response from the host to the dysbiotic microbial biofilm [75]. However, some periodontal bacteria also play a direct role in the loss of gingival epithelial integrity. Recent work has highlighted that gingipains secreted by *P. gingivalis* degrade tight junction-associated proteins, resulting in the permeability of the gingival epithelium to gingipains, LPS and proteoglycans [76]. These findings are in agreement with previous results from Katz et al. [77], showing that the same proteases degrade epithelial junction proteins such as E-cadherin, occludin and β1-integrin. This loss of integrity may contribute to the translocation of bacterial components into the bloodstream and periodontium distant tissues. Concordantly, in humans, periodontal bacteria DNA has been detected in abdominal aortic aneurysm, atherosclerotic plaques and aortic tissue [78,79,80]. In vivo, periodontitis models of the oral application of *P. gingivalis* in mice exhibit alveolar bone resorption associated with the detection of *P. gingivalis* bacteria and gingipains in the brain [81] and pancreas [82].

### 3.2. Impact of Periodontal Bacteria on Insulin Sensitivity and Secretion

The presence of a periodontal infection elevates the plasma levels of TNF-α, IL-6 and C-reactive protein (CRP) [83,84] and insulin resistance markers such as HOMA-IR [85] in periodontitis patients. Moreover, periodontitis is associated with increased oxidative stress as evidenced by lower plasma small molecule antioxidant capacity (pSMAC) and higher levels of plasma protein oxidation in type 2 diabetic patients [86]. Interestingly, numerous studies report that the therapeutic management of periodontitis is associated with reduced glycated hemoglobin (HbA1c) and systemic inflammation and improved glycemic control in diabetic patients with periodontitis [87,88,89].

To investigate the link between periodontal disease and insulin resistance, various in vivo models have been developed. These models include the oral application of whole periodontal bacteria [90] or LPS to the gingival sulci [91], silk ligature with [92,93,94] or without [95] the application of periodontal bacteria components, and intravenous injection of sonicated bacteria [96]. Each of these models exhibits insulin resistance and impaired glucose metabolism, which was demonstrated by a glucose tolerance test (GTT) and insulin tolerance test (ITT), and systemic inflammation characterized by elevated plasma IL-6 [11] and TNF-α [95] levels. Watanabe et al. [93] reported that periodontitis accelerates the onset of severe insulin resistance and impaired glucose homeostasis in Zucker diabetic fatty rats. Interestingly, Blasco-Baque et al. [12] demonstrated that adaptative immune response developed against *P. gingivalis* prior to periodontal infection protects mice from the deleterious effects of periodontitis on glucose tolerance.

Considering these profound metabolic alterations, it is essential to understand the molecular mechanisms linking insulin resistance and periodontal bacteria by focusing on insulin-dependent tissues, including adipose tissue, skeletal muscle, the liver and pancreatic β-cells responsible for insulin secretion.

#### 3.2.1. Adipose Tissue

The deregulation of adipose tissue physiology plays a central role in various diseases, including cardiovascular diseases and diabetes. Obesity is strongly associated with diabetes [2]. During obesity, excessive fat accumulation in adipocytes impairs their secretory and metabolic functions. Adipose cells overproduce pro-inflammatory adipokines, leading to a chronic low-grade inflammatory state [53]. Obesity also promotes the overproduction of ROS and a deficit in endogenous antioxidant defense, leading to oxidative stress [58]. These disorders contribute to the development of insulin resistance and type 2 diabetes.

The literature data report a positive correlation between obesity and the prevalence of periodontal diseases [97]. During obesity, periodontal bacterial components may induce chronic inflammation and oxidative stress, contributing to the development of insulin resistance. Of note, a mouse model co-exposed to high-fat diet (HFD)-induced obesity and experimental periodontitis exhibits worsened inflammation and insulin resistance compared to obese mice without periodontitis [12].

There is still no evidence that periodontal bacteria can reach adipose tissue during periodontitis. However, a previous in vivo study demonstrated that oral administration of *P. gingivalis* in mice induces insulin resistance associated with macrophage infiltration on epididymal adipose tissue. Moreover, this tissue exhibited upregulation of the expression of genes encoding TNF-α, IL-6, MCP-1 and IL-1β, and in parallel, downregulation of the expression of genes coding for IRS-1 and Sirt1 involved in insulin sensitivity [11]. A decrease in IRS-1 phosphorylation involved in the insulin signaling pathway was also reported in the adipose tissue of rat models with ligature-induced periodontitis [95].

In vitro studies on the murine 3T3-L1 adipose cell line have further investigated the impact of periodontal bacteria exposure on adipocyte metabolism. In this cell line, *P. gingivalis* bacteria exert pro-inflammatory action by activating TLR2/4 receptors and recruitment of NF-κB, p38 MAPK, JNK and extracellular signal-regulated kinase (ERK) signaling pathways [49,98]. These signaling pathways promote increased secretion of the pro-inflammatory cytokines IL-6, MCP-1, TNFα and leptin and decreased release of adiponectin. Accordingly, Yamaguchi et al. [99] reported a dose-dependent increase in IL-6 secretion in response to LPS of *P. gingivalis* and *Fusobacterium nucleatum* in 3T3-L1 adipocytes.

In the study by Le Sage et al. [49], we demonstrated that the exposition of 3T3-L1 adipocytes to *P. gingivalis* LPS induces oxidative stress by increasing intracellular ROS levels and altering the expression of genes encoding redox enzymes. In particular, *P. gingivalis* LPS enhances the expression of genes coding for NOX2, NOX4, iNOS and the antioxidant enzyme catalase, suggesting an activation of the antioxidant defense system in response to oxidative stress. Singh et al. [98] reported similar results during adipocyte exposure to *P. gingivalis* whole bacteria and demonstrated downregulation of the expression of genes encoding heme oxygenase-1 (HO-1) and peroxisome proliferator-activated receptor gamma coactivator 1-alpha (PGC1-α). Interestingly, previous studies reported that the suppression of HO-1 gene expression in adipose tissue is associated with a decrease in PGC1-α, leading to mitochondrial dysfunction and increased inflammation [100].

In 3T3-L1 adipocytes, *P. gingivalis* LPS also alters adipogenic and insulin sensitivity-related markers. Indeed, *P. gingivalis* LPS exposure leads to increased secretion of resistin and leptin involved in insulin resistance and decreased secretion of adiponectin recognized as an insulin-sensitizing molecule [49]. In mice isolated primary adipocytes, infection with *P. gingivalis* reduces levels of the insulin receptor and Akt phosphorylation [99]. *P. gingivalis* also increases the mRNA levels of CCAAT/enhancer-binding protein alpha (CEBPα), fatty acid synthase (FAS) and peroxisome proliferator-activated receptor gamma (PPARγ), and enhances the accumulation of lipid droplets in 3T3-L1 adipocytes [98].

Together, these studies support the deleterious impact of periodontal bacteria on adipocyte metabolism, which may contribute to the development of insulin resistance associated with periodontitis, and link obesity to type 2 diabetes and periodontal disease.

#### 3.2.2. Skeletal Muscle

In humans, anti-*P. gingivalis* antibody tiers are positively correlated with intramuscular adipose tissue content (IMAC), fasting blood glucose and HOMA-IR insulin resistance index [13]. However, to date, there are little data regarding the impact of periodontitis on skeletal muscle tissue and glucose metabolism.

Watanabe et al. [13] demonstrated impaired glucose tolerance, insulin resistance and marked fat infiltration in skeletal muscles of C57BL/6J mice fed an HFD and exposed to *P. gingivalis* by oral administration (HFPg) when compared to control animals (HFco). The soleus muscle of HFPg mice exhibits fat infiltration and lower glucose uptake resulting from impaired insulin signaling by decreased Akt phosphorylation. These alterations were associated with higher expression levels of TNF-α, IL-6 and MCP-1, enrichment of gene sets including the IL-6/JAK/STAT pathway, and gene sets related to TNF-α signaling via NF-κB. Concordantly, in vitro exposition of C2Cl2 myoblasts to TNF-α decreases glucose uptake. In parallel, in mice fed a normal chow diet, administration of *P. gingivalis* also induces insulin resistance associated with decreased Akt phosphorylation and increased TNF-α expression in the soleus muscle.

Even if there is currently no data regarding the impact of periodontal components on muscle cells’ insulin pathway, a previous study demonstrated that the exposition of human muscle cells to *Escherichia coli* LPS increased MCP-1 and IL-6 gene expression and JNK phosphorylation. Consequently, insulin-stimulated IRS-1, as well as Akt and AS160 phosphorylation, were reduced. Interestingly, this deleterious impact of LPS was counteracted by using a TLR4 antagonist [65]. These results raise the possibility of the same impact of periodontal bacteria LPS, which mediate pro-inflammatory action via TLR4 activation.

#### 3.2.3. The Liver

A growing number of studies show the involvement of periodontitis in the dysregulation of liver metabolism and progression of liver diseases [101,102]. In vivo studies have demonstrated that periodontitis increases fat accumulation and exacerbates non-alcoholic fatty liver disease (NAFLD) in mouse livers. Sasaki et al. [96] reported increased liver steatosis with elevated triglycerides and glycogen accumulation in HFD-exposed mice when intraperitoneally administered with sonicated *P. gingivalis*. Similar results were obtained in HFD-induced obese rats with periodontal ligature [94] or orally administrated with *P. gingivalis* [11]. This fat accumulation is associated with a significant increase in hepatic CD36 mRNA levels [94,96]. Hepatic CD36 is a fatty acid transporter that acts as a transcriptional regulator of PPARγ. Its depletion from hepatocytes attenuates fatty liver and improves insulin sensitivity in obese animals [103]. Accordingly, it was demonstrated that *P. gingivalis* exacerbates the progression of fatty liver disease through the CD36-PPARγ pathway [104]. Interestingly, Ni et al. [94] showed that periodontal scaling and root planing decrease the mRNA levels of hepatic CD36 and CRP levels and improve insulin resistance in obese rats. In addition to altered lipid metabolism and enhanced fatty liver disease, periodontal bacteria also impact glucose metabolism. In vitro studies using HepG2 human hepatocytes treated with *P. gingivalis* showed that the bacteria internalizes into hepatocytes and reduces glycogen synthesis by attenuating the phosphorylation of IRS-1 and the Akt/glycogen synthase kinase-3β (GSK-3β) signaling pathway [105]. Similarly, Seyama et al. [18] recently demonstrated that *P. gingivalis* OMVs carrying gingipains translocate into the liver and attenuate glycogen synthesis in mice. Exposing HepG2 hepatocytes to these OMVs provided evidence for an attenuation of the insulin-induced Akt/GSK-3β signaling pathway.

In the liver, *P. gingivalis* bacteria induce increased production of IL-6 and TNF-α and upregulate gene sets related to TNF-α signaling via NF-κB [11,96]. The same effect is observed in in vitro Hepa-1.6 hepatocytes cell line in response to *P. gingivalis* exposure [106]. Of note, in experimental mouse models of periodontitis, TLR4 loss of function inhibits the deleterious effect of periodontitis on the insulin signaling pathway, evidenced by the increased ratio of pAkt/Akt and decreased levels of TNF-α [92].

Metabolomic analyses of the liver of animals with an oral application of *P. gingivalis* revealed a marked increase in biomarkers of oxidative stress, such as methionine sulfoxide and S-methylcysteine [90], suggesting that *P. gingivalis* also deregulates redox homeostasis, resulting in the production of free radicals and peroxides that may contribute to inflammation and insulin resistance in the liver.

#### 3.2.4. Pancreatic β-Cells

The presence of periodontal bacteria from *Fusobacterium* species has been reported in pancreatic cancer [107]. Recent data from Ilievski et al. [82] demonstrated the presence of *P. gingivalis* bacteria and gingipains in pancreatic β-cells in both human and animal models of periodontitis, indicating that periodontal bacteria translocate into pancreatic islets. However, while in mice, gingipains are found only in β-cells, in humans, they are also found in α-cells at a lower proportion. In animal models, periodontitis induction is associated with hyperinsulinemia [90], suggesting a direct impact of periodontal bacteria on the function of pancreatic β-cells, the only known source of circulating insulin. In the murine pancreatic β-cell line MIN6, *P. gingivalis* and its LPS induce an increase in insulin secretion [108] associated with the elevation of SerpinE1 gene expression [109]. The serpinE1 gene encodes the plasminogen activator inhibitor-1 (PAI-1), which is known to increase during obesity, insulin resistance and diabetes [110].

The translocation of *P. gingivalis* in the pancreatic islet is associated with changes in islet architecture, upregulation of SerpinE1 and β-cells apoptosis [111]. Whereas in control animals exposed to *P. gingivalis*, α-cells are only located in the mantle zone surrounding the β-cell core, in experimental animals, α cells are found in the mantle zone as well as inside the β-cell core. Consistent with this observation, in vitro exposure to *P. gingivalis* induces apoptosis of MIN6 β-cells, which is reduced by SerpinE1 inhibition. In the study by Ilievski et al. [82], it was demonstrated that gingipains that translocate into β-cells in human pancreatic samples and experimental mice are located at the nuclear or peri-nuclear levels. Moreover, the presence of bihormonal cells was detected in pancreatic islets. It was found that these bihormonal cells are more prevalent in diabetic patients and positively correlate with *P. gingivalis* invasion in both human and mouse samples. Interestingly, only a low proportion of these bihormonal cells show an intracellular presence of gingipains. Bihormonal cells are glucagon and insulin-producing cells and are considered to be at an intermediate α- to β-cell differentiation step [112]. The emergence of bihormonal cells has already been reported following the near-total loss of β-cells in animal models [113]. In parallel, in humans, a higher percentage of bihormonal cells has been reported in pancreatic samples from an insulin-resistant group, as compared to an insulin-sensitive group [114]. This process is thought to be adopted to compensate for insulin resistance and insulinopenia.

## 4. Current Management of Periodontal Infection and Diabetes

Since daily home care enables dental biofilm removal, establishing an effective oral care routine is an essential component of periodontal infection prevention and treatment. This should be associated with the correction of aggravating factors [115]. The objective of periodontal treatment is to mechanically remove bacterial deposits and calculus from the subgingival plaque to stop periodontitis and, when possible, to regenerate the periodontium lost as a result of the disease. This treatment can be surgical or non-surgical, according to the severity of periodontal lesions. Non-surgical treatments consist of scaling and root planing and allow the removal of supra- and subgingival calculus to facilitate the re-attachment of the gums to the roots. Systemic antibiotics can also be prescribed as a supplement and are associated with significantly improving scaling and root scaling outcomes [116]. However, for the most advanced periodontitis with deeper periodontal pockets (i.e., 6 mm or deeper) and bone lesions, periodontal surgery needs to be performed. Surgical therapies include resective surgery to reduce and eliminate inflammatory lesions and regenerative surgery, such as guide tissue regeneration and bone grafts, to re-establish lost periodontal tissues [117]. Guide tissue regeneration is accomplished using a barrier membrane with or without bone grafting materials. Various biological factors can also be used to enhance the outcome of periodontal regeneration. Systematic review and meta-analysis reported greater clinical attachment when certain biological factors are added to regenerative treatment. This is the case for enamel matrix derivatives, which promote bone formation, periodontal ligament cell proliferation [118], and recombinant human platelet-derived growth factor-BB (PDGF-BB), a wound healing growth factor [119]. Interestingly, a recent randomized trial showed that intensive periodontal treatment, consisting of whole mouth subgingival scaling and surgical periodontal therapy, reduced fasting plasma glucose concentrations, reduced systemic inflammation, and improved vascular and kidney function as well as quality of life [87].

Currently, managing diabetes includes lifestyle changes with a nutritional approach and regular practice of physical exercise and pharmacological therapies when lifestyle measures alone are unable to sustain glycemic control. Various pharmaceutical compounds with different modes of action are available to treat type 2 diabetes [120,121]. Insulin-sensitizers, such as biguanides and thiazolidinediones, act by improving tissue sensitivity to insulin, while insulin secretagogues, such as meglitinides and sulfonylureas, stimulate insulin secretion by pancreatic β-cells. Another class of antidiabetics includes glucagon-like peptide-1 (GLP-1) receptor agonists, which mimic GLP-1 activity, and inhibitors of dipeptidyl peptidase-4 (DPP-4) [122]. Indeed, the incretin hormone GLP-1 exerts antihyperglycemic activity by regulating appetite and satiety, inhibiting glucagon secretion and promoting insulin production but is rapidly degraded by DPP-4 in physiological conditions. Inhibitors of α-glucosidase aim to limit carbohydrate digestion and absorption in the intestinal tract and reduce postprandial hyperglycemia [123]. Lastly, inhibitors of sodium-glucose co-transporter (SGLT)-2 facilitate the excretion of glucose in the urine by inhibiting its reabsorption in the proximal renal tubules [124]. In the gastrointestinal tract, SGLT-1 is responsible for glucose and galactose absorption. A dual SGLT1/2 inhibitor has already demonstrated promising efficacy for controlling glycemia [125]. Importantly, in patients with advanced type 2 diabetes, these medications fail to effectively regulate hyperglycemia, and these patients have to be placed on insulin replacement therapy.

Although current therapies for type 2 diabetes control hyperglycemia and reduce diabetes symptoms, they also exert undesirable side effects. Moreover, they do not target the molecular mechanisms responsible for the development of insulin resistance and related diabetes, including inflammation and oxidative stress. Thus, there is very high interest in validating innovative anti-inflammatory and antioxidant strategies such as those using plant polyphenols.

## 5. Polyphenol-Based Therapies

### 5.1. Structures and Sources of Polyphenols

Polyphenols are defined as plant secondary metabolites with nutritional and pharmacological potentials. They are naturally found in different plant compartments such as flowers, leaves, stems, barks and roots, and different sources including fruits, vegetables, cereals, medicinal plants and derived beverages, in variable proportions [126]. Plant polyphenols are generated from primary metabolites and intermediates thanks to biosynthetic pathways [127]. They are involved in the defense system of plants against different types of environmental stress like ultraviolet rays and pathogenic attacks, and plant development and growth. In addition, polyphenols are responsible for the color, smell and taste of plants, such as bitterness and astringency [126]. These phenolic compounds are the most abundant antioxidant micronutrients the human diet provides. In most cases, food contains complex mixtures of polyphenols, but some classes of phenolic compounds are specific to certain plants.

**Figure 3:**
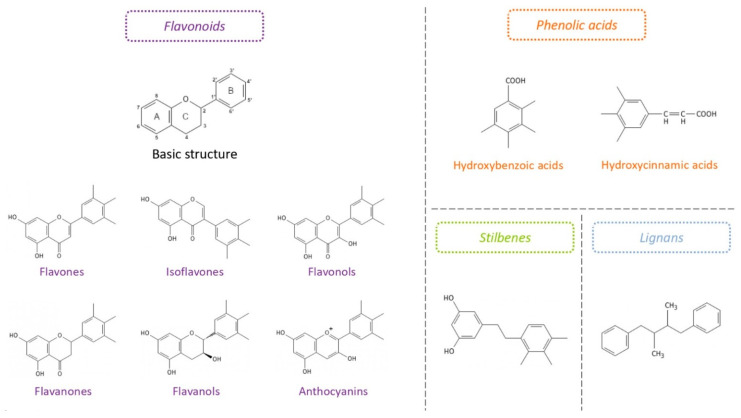
Structural classification of dietary polyphenols. Dietary polyphenols are classified into 4 major chemical families, including flavonoids, phenolic acids, stilbenes and lignans. The flavonoids are subdivided into 6 subclasses named flavones, isoflavones, flavonols, flavanones, flavanols and anthocyanins, which share a common structure composed of 2 aromatic rings (**A**,**B**) bound together by 3 carbon atoms forming an oxygenated heterocycle (**C**). The phenolic acids comprise hydroxybenzoic and hydroxycinnamic acids, derivatives of benzoic and cinnamic acids, respectively. The stilbenes are composed of 2 phenyl groups joined together by a methylene bridge. The lignans are composed of 2 phenylpropane units.

Chemically, polyphenols are characterized by a structure comprising one or several aromatic rings with at least one hydroxyl (OH) group able to neutralize a free electron while remaining stable. This phenolic structure gives them an antioxidant property. Rice-Evans et al. [128] showed a relationship between the structure of polyphenols and their antioxidant capacity. In particular, the polyphenols carrying an aromatic nucleus with two OH groups (catechol group) may exert stronger antioxidant effects than those with only one OH group. Currently, nearly 8000 polyphenols have been identified and classified into major chemical families according to the structure of their carbon skeleton and the number of phenolic rings (Figure 3). There are the phenolic acids, comprising hydroxybenzoic acids (derivatives of benzoic acid) and hydroxycinnamic acids (derivatives of cinnamic acid); the flavonoids that gather more than 5000 molecules, which share a common structure formed by two aromatic rings bound together by three carbon atoms forming an oxygenated heterocycle subdivided into different subclasses called flavones, isoflavones, flavonols, flavanones, flavanols (monomers, proanthocyanidin polymers) and anthocyanins; the stilbenes, with two phenyl groups joined together by a methylene bridge; and the lignans, with two phenylpropane units [129].

Concerning the phenolic acids, edible plants contain few hydroxybenzoic acids, whether in free or esterified forms. However, they are found in large quantities in hydrolyzable tannin forms such as ellagitannins detected in certain red fruits such as strawberry (20–90 mg/kg fresh weight) or blackberry (80–270 mg/kg fresh weight) [130]. They are also found in onions and tea leaves containing molecules such as gallic acid in a significant quantity (4.5 g/kg fresh weight) [129]. Otherwise, among the hydroxycinnamic acids, the most frequently found compound is caffeic acid, which alone accounts for 75–100% of the total hydroxycinnamic acids from most fruits. Caffeic acid is abundant in cereals (0.8–2 g/kg in wheat grains), certain vegetables such as eggplant (600–660 mg/kg), and kiwi (0.6–1.0 g/kg fresh weight), coffee beans, peanuts, apples, oranges and pineapples. Hydroxycinnamic acids are generally present as glycosylated derivatives or esters [129]. Chlorogenic acid, an ester of caffeic and quinic acids, is the most common conjugate found at very high concentrations in many fruits and coffee (a cup of coffee can contain 50 to 150 mg). The methylated derivative of caffeic acid called ferulic acid is mainly present in wheat seeds (0.8–2 g/kg weight). Furthermore, the most ubiquitous dietary flavonoids are flavonols, particularly quercetin and kaempferol, generally found at concentrations ranging from 15 to 30 mg/kg fresh weight in onions (up to 1.2 g/kg fresh weight), curly kale (300–600 mg/kg fresh weight), leeks (30–225 mg/kg fresh weight) and blueberries (250–5000 mg/kg fresh weight). According to Scalbert and Williamson [126], the daily consumption of polyphenols is estimated at around 1 g with major consumption of flavonoids (60%) comprising 460 mg of proanthocyanidins, 200 mg of catechins, 180–215 mg of anthocyanins and 115 mg of flavones and flavonols [126,131]. Stilbenes are found in low quantities in the human diet, except for resveratrol, widely present in various quantities in dark chocolate (350 μg/kg), red grapes (92–1604 μg/kg fresh weight) and white grapes (59–1759 μg/kg fresh weight) [132]. Linseed is the richest dietary source of lignans containing secoisolariciresinol (up to 3.7 g/kg dry weight) and low quantities of matairesinol [133]. Notably, our previous studies demonstrate the abundance of different polyphenols comprising caffeic acid esters, quercetin and kaempferol glycosylated derivatives, and ellagitannins or curcuminoids in various sources such as tropical fruits [134], medicinal plants [135] and *Curcuma longa* turmeric [136].

### 5.2. Bioavailability of Polyphenols

The biological properties of dietary polyphenols are strongly dependent on their bioavailability, namely their extent of absorption, distribution, metabolism and elimination. The bioavailability extent of polyphenols and related metabolites governs their ability to reach target tissues and exert local biological effects. Three major factors modulate the polyphenol bioavailability, establishing the nature and concentrations of the metabolites circulating in the body and target tissues. They are (i) the rate of intestinal absorption, (ii) the magnitude of metabolism by enterocytes and hepatocytes, and (iii) the degradation extent by the gut bacterial microbiota [137,138]. Notably, polyphenols found in foods are not necessarily those that lead to the most active metabolites in the target tissues. Indeed, chemical structure, concentration, nature of metabolites, rate and degree of absorption are important parameters to take into account [139]. Polyphenol’s biological properties greatly differ from one polyphenol to the next. The process of polyphenol bioavailability begins when the polyphenols are released from the food matrix, absorbed through the intestinal barrier, deconjugated in the gastrointestinal tract and colon, and then transported into the bloodstream to reach the target tissue. Of the total intake of polyphenols ingested through food in the form of esters, glycosides or polymers, only 5 to 10% can be directly absorbed in the form of aglycones in the small intestine after deconjugation reactions, such as deglycosylation by bacterial glycosidases and esterases, before being transported in a less complex form to the portal vein [129]. There are two enzymes essential for the hydrolysis, release and transport of aglycones, namely lactase-phlorizin hydrolase and cytosolic β-glucosidase, found in enterocytes of the small intestine [140]. Subsequently, the metabolites formed and the catabolites originating from colonic microbial metabolism are metabolized in the liver. Indeed, polyphenol metabolites may undergo phase I associated with oxidation, reduction and hydrolysis reactions, followed by phase II conjugation characterized by reactions such as methylation (in the gut), sulfation (in the liver) and glucuronidation (in both the gut and the liver) [141]. The conjugation reactions depend on the nature of the substrates and the dose of polyphenols ingested. In addition, the biological properties of polyphenols may depend on the degree of conjugation/deconjugation of polyphenols at the hepatic level [141]. When they leave the liver, a part of the tissue metabolites can be excreted in the bile and undergo an enterohepatic cycle transporting them back to the small intestine. When polyphenols are not absorbed and metabolized in the intestine, they enter the colon and are degraded by colonic bacteria [127,138,141]. We contributed to identifying several microbial metabolites of polyphenols that include derivatives of phenylvaleric, phenylpropionic, phenylacetic and phenylbenzoic acids, with evidence of a high level of hippuric acid [142,143,144]. Interestingly, Brial et al. [145] recently reported that hippuric acid administration improved glucose tolerance and insulin secretion in mice exposed to HFD-induced obesity. A positive link between the urinary concentration of hippuric acid and glucose homeostasis was established in volunteers consuming a high-meat diet rich in saturated fats, highlighting hippuric acid as a new mediator and biomarker of metabolic health. In parallel, some specific microbial metabolites originating from the catabolism of certain types of polyphenols have been described, such as equol deriving from soy isoflavones [146] or urolithins from ellagic acid and ellagitannins abundant in pomegranate [147]. If they are not used, the low molecular weight metabolites are eliminated in the bile or urine [129,142]. Currently, data concerning the bioavailability of polyphenol metabolites in tissues are still very scarce. However, polyphenols such as quercetin, epigallocatechin gallate, resveratrol, caffeic acid and ferulic acid have been detected in a wide range of tissues in mice and rats, including the brain, endothelial cells, heart, kidney, stomach, intestine, liver, spleen, pancreas, prostate, uterus, ovaries, mammary glands, testes, bladder, bone and skin [139,148,149,150,151,152] in low concentrations, depending on the dose administered and the tissue considered. Considering that the kinetics of penetration and elimination of polyphenols or metabolites in tissues remain poorly understood, it is important to determine the suitable time of tissue sampling.

Recently, some authors reported an important notion regarding polyphenol bioavailability, namely the crucial impact of gut bacterial microbiota, which makes it possible to define people who produce or do not produce a particular type of microbial metabolite. They showed the need, in nutritional interventions, to better consider the existence of different types of metabolism or “metabotypes” that depend on interindividual variability in terms of the absorption, metabolism and biological effects of polyphenols [153,154]. Nowadays, robust analytical tools such as mass spectrometry can provide the metabolomic fingerprint associated with consuming a particular type of polyphenol or food. Thus, biomarkers of consumption of/exposure to polyphenols are sought to correlate their biological effects [155,156]. The bioavailability rate already depends on the polyphenol amount consumed and the plasma concentration found [137]. Notably, polyphenol plasma concentration rarely exceeds 1 µM despite reaching 7–10 µM for some polyphenols, depending on the interindividual variability of absorption and metabolism related to gut microbiota composition, the type of polyphenol, the ingested dose and food source [153,154].

### 5.3. Biological Effects of Polyphenols

The consumption of polyphenol-rich foods may play a role in preventing certain pathologies such as cancer, osteoporosis, neurodegenerative diseases, type 2 diabetes and cardiovascular diseases [157]. Polyphenols may exert pleiotropic health benefits, but they are of particular interest in managing type 2 diabetes due to their anti-inflammatory, antioxidant, insulin-sensitizing and anti-bacterial properties [19].

#### 5.3.1. Anti-Inflammatory Properties of Polyphenols

The anti-inflammatory effect of polyphenols is widely described in the literature. Polyphenols can reduce the production of various pro-inflammatory cytokines and chemokines, such as IL-6, IL-8, TNF-α and MCP-1, due to their ability to inhibit NF-κB and MAPK signaling pathways [158,159,160]. Mice fed with curcumin experienced less massive macrophage infiltration in the adipose tissue and inhibited NF-κB pathway, increased adiponectin production, and decreased hepatic NF-κB activation induced by HFD [161]. Many existing studies display the anti-inflammatory properties of polyphenols, including resveratrol [162], quercetin [163] and tea [164].

#### 5.3.2. Antioxidant Properties of Polyphenols

Polyphenols were first described for their potent antioxidant capacity, which may strengthen cellular defense against oxidative stress and its consequences. It is suggested that the antioxidant properties of polyphenols, linked to their free radical-scavenging ability and antioxidant enzyme modulation capacity, could reduce oxidative stress. Several dietary polyphenols, including resveratrol, quercetin, tea catechins and curcumin, upregulate the redox-sensitive transcriptional factor Nrf-2 and several antioxidant enzymes (SOD, catalase and GPx), which downregulate ROS production and limit oxidative stress [159,165]. Polyphenols are able to target various proteins, including enzymes, receptors and transporters both at the cytosolic and mitochondrial levels, keeping in mind that mitochondria constitute a major source of ROS. The inhibition of free radical-generating enzymes such as NOX is an important mechanism of the antioxidant effect for polyphenols. Several works have reported that flavonoids are the molecules most likely involved in this effect by inhibitor-enzyme complex formation and/or by direct scavenging of ROS [166].

#### 5.3.3. Insulin-Sensitizing Properties of Polyphenols

Numerous studies highlight the anti-diabetic properties of various polyphenolic compounds. Anthocyanins present in bilberries and other berries enhance the insulin secretion of pancreatic β-cells and insulin sensitivity of 3T3-L1 adipocytes [167,168]. In db/db or HFD-induced obese and diabetic mice models, resveratrol and curcumin lowered fasting blood glucose levels and increased insulin sensitivity. These polyphenols were also able to raise the level of phosphorylated AMPK in skeletal muscles leading to improved insulin response and preserving pancreatic β-cell mass against oxidative stress and elevated pancreatic insulin content [169,170,171,172,173]. While the insulin-sensitizing properties of polyphenols are promising on cell and animal models, results from clinical trials remain controversial. In a meta-analysis, Raimundo et al. [174] reviewed the effects of polyphenol intervention in human randomized controlled trials. Briefly, the main polyphenols used in human studies are green tea catechins, resveratrol, curcumin and quercetin. Polyphenol consumption lowered fasting blood glucose levels in type 2 diabetic patients, except for curcumin. Nevertheless, curcumin was able to decrease the risk of developing type 2 diabetes and improved HOMA-IR values and pancreatic β-cell function in prediabetic patients [175]. In patients with prediabetes or diabetes, curcuminoid treatment for three months improved fasting blood glucose, HbA1c levels and insulin resistance [176,177]. Overall, the literature data suggest that polyphenol interventions in clinical trials are promising for lowering blood glucose and could enhance insulin sensitivity. However, clinical and preclinical studies are still required to better understand the mechanisms underlying polyphenol benefits for diabetes management and to elucidate controversial data.

#### 5.3.4. Anti-Bacterial Properties of Polyphenols

A close link between gut microbiota dysbiosis, obesity and type 2 diabetes has been reported. A prominent role of gut microbiota dysbiosis in contributing to inflammation, oxidative stress and insulin resistance was evidenced [178]. Given their various beneficial health properties, such as those described above, polyphenols could act efficiently against gut microbiota dysbiosis due to their anti-bacterial properties. Beyond their anti-bacterial effect, polyphenols act specifically against bacterial pathogens, while beneficial bacteria remain unaffected if not stimulated. Tzounis et al. [179] demonstrated that epicatechin and catechin increased the count of probiotics and inhibited foodborne pathogens. In parallel, the newly emerging concept is related to the prebiotic properties of polyphenols able to shape the gut microbiota composition and attenuate gut dysbiosis during obesity and diabetes [180]. For a comparative analysis of polyphenols with other plant metabolites, prebiotic activities have been largely attributed to some dietary fermentable fibres or non-digestible oligosaccharides that act as non-viable substrates and serve as nutrients for selective beneficial microorganisms within the gut microbiota, conferring a net health benefit [181]. In the recent study by Belda et al. [182], we found that supplementation with fructo-oligosaccharides and biotin (vitamin B8) in high-fat diet-fed mice improved gut microbial diversity and bacterial production of biotin and other B vitamins and reduced fat mass gain, fasting glycemia and the HOMA-IR index indicating insulin resistance. Concerning polyphenols, it is also suggested that phenolic metabolites, especially ones with an aromatic ring in their structure resulting from the gut bacterial transformation of polyphenols, often exhibit different and/or enhanced biological activity than their parent forms [183]. Due to anti-bacterial properties that promote beneficial bacteria growth, polyphenols could also be used to manage periodontitis resulting from oral dysbiosis.

### 5.4. Polyphenol-Based Therapy for Periodontitis Management

**Figure 4:**
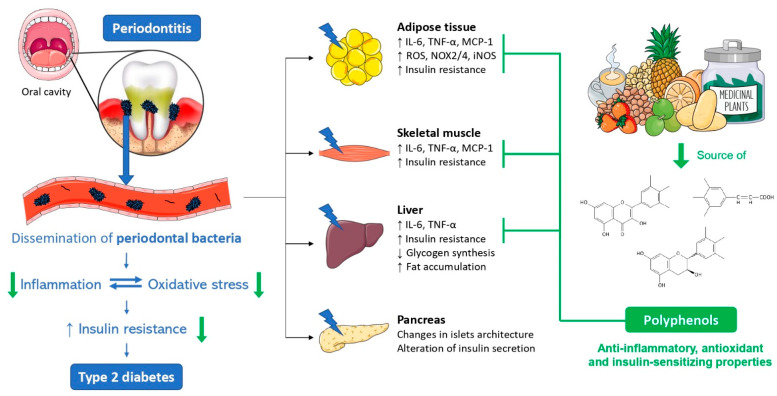
Overview of periodontal bacteria detrimental effects related to insulin resistance and therapeutic potential of polyphenols. Periodontitis results from oral microbiota dysbiosis and leads to the progressive destruction of the periodontal tissues. During periodontitis, periodontal bacteria components disseminate into systemic circulation and may reach distant tissues, including adipose tissue, skeletal muscle, liver and pancreas. Locally, periodontal bacteria enhance the production of pro-inflammatory cytokines such as IL-6, TNF-α and MCP-1 and promote oxidative stress. Crosstalk between players of inflammation and oxidative stress induces insulin resistance. Moreover, in the liver, periodontal bacteria alter glucose and lipid metabolism by decreasing glycogen synthesis and increasing fat accumulation. In the pancreas, periodontitis is associated with changes in the islets’ architecture and alteration of insulin secretion. Altogether, these detrimental effects of periodontal bacteria contribute to type 2 diabetes. Interestingly, polyphenols able to exert anti-inflammatory, antioxidant, insulin-sensitizing and anti-bacterial properties may help to counteract the deleterious action of periodontal bacteria and improve insulin resistance.

Current periodontal treatments are invasive and do not allow a basal return of the inflammatory and metabolic status of diabetic patients. Accumulating evidence highlights the use of polyphenols as an innovative natural therapy to improve oral dysbiosis-worsened pro-inflammatory and metabolic status and insulin sensitivity in diabetic patients. Concerning oral dysbiosis, resveratrol can reduce *P. gingivalis* LPS-induced TNF-α, IL-6 and IL-1β production in human periodontal ligament cells aggravating destructive tissue processes in periodontitis and promoting systemic inflammation [184]. Concerning periodontal bacteria and insulin sensitivity, we previously showed that medicinal plant polyphenols increased the production of adiponectin and PPARγ, key anti-inflammatory and insulin-sensitizing mediators, and exerted antioxidant properties by reversing oxidative stress on adipose cells exposed to *P. gingivalis* LPS [185]. Therefore, polyphenols could act (1) locally to treat periodontitis by favoring bacterial communities and downregulating inflammation, and (2) downstream or through phenolic metabolites to counteract inflammatory and metabolic disorders during diabetes. Figure 4 proposes an overview of the detrimental effects of periodontal bacteria on key tissues related to insulin resistance, including the adipose tissue, skeletal muscle, liver and pancreas, and the therapeutic potential of plant-derived polyphenols able to exert anti-inflammatory, antioxidant and insulin-sensitizing activities. However, there is still a lack of evidence regarding the benefits of polyphenols against periodontitis during obesity/diabetes. Likewise, the evaluation of polyphenol’s effects on glucose-related and insulin-dependent organ functions, such as the liver, pancreas, adipose tissue and skeletal muscle, remains to be investigated.

As mentioned above, polyphenols are recognized as the most abundant antioxidants provided by the human diet. Interestingly, other natural micronutrients, including carotenoids and vitamins, exhibit antioxidant properties and act as anti-inflammatory mediators. Their actions may synergize with those of polyphenols in nutritional contexts or as additional potential therapeutic agents. Regarding the carotenoids, β-carotene, serving as a precursor of vitamin A, has been reported to suppress *P. gingivalis* LPS-induced pro-inflammatory cytokine production by monocytes cultured in high glucose conditions [186]. The same effect was detected in human periodontal ligament cells treated with β-cryptoxanthin [187]. In a rat model of experimental periodontitis, treatment with fucoxanthin led to a decrease in blood levels of TNF-α and IL-6 [188]. Concerning the vitamins, vitamin E, also known as α-tocopherol, reduced the secretion of pro-inflammatory cytokines and increased cell proliferation and migration in human gingival fibroblasts [189]. Vitamin C was also reported to provide protective effects against inflammation and oxidative stress in periodontal tissues of the ligature-induced periodontitis rat model [190]. Nevertheless, similar to the effects of polyphenols, the protective role of compounds such as carotenoids or vitamins E and C on insulin-targeted tissues and insulin resistance associated with periodontal disease still needs to be elucidated.

## 6. Conclusions

The growing literature data support a bidirectional link between diabetes and periodontitis. During periodontitis, periodontal bacteria translocate into systemic circulation and reach distant tissues, resulting in increased systemic inflammation and insulin resistance. In the adipose tissue, skeletal muscle and liver, these bacteria activate pivotal players of inflammation and oxidative stress by elevating the secretion of pro-inflammatory cytokines including IL-6, TNF-α, MCP-1 and redox markers. The activation of such molecular mediators promotes the disruption of the insulin signaling pathway, mainly by serine phosphorylation of IRS-1 that leads to insulin resistance and a glucose uptake blockade. In addition, periodontal bacteria alter insulin secretion by pancreatic β-cells, aggravating insulin signaling deregulation and hyperglycemic status.

Periodontal bacteria may also contribute to insulin resistance through gut microbiota modifications. Indeed, several studies reported that periodontitis alters gut microbiota [11,12,96]. Nakajima et al. [191] demonstrated that oral administration of *P. gingivalis* in mice induces gut microbiota dysbiosis characterized by the enhanced proportion of the *Firmicutes* phylum and reduced proportion of the *Bacteroidetes* phylum. This was associated with increased serum endotoxin levels and decreased expression of intestinal tight junction proteins. Given the already well-described link between intestinal bacteria and insulin resistance [178], swallowed periodontal bacteria-induced microbiota dysbiosis may be another mechanism linking periodontitis and type 2 diabetes.

Therapeutic management of periodontitis improves the systemic inflammation and insulin resistance markers in diabetic patients with periodontitis [192]. Thus, establishing a daily home care routine and regular dental care should be integrated with type 2 diabetes prevention and treatment. Moreover, severe periodontitis is associated with elevated insulin resistance markers in non-diabetic subjects who have a higher probability of developing type 2 diabetes when compared to periodontally healthy people [8,85,193]. This suggests that the presence and severity of periodontitis may be involved in the early detection of diabetes and could serve as useful clinical biomarkers. Thus, it will be interesting to institute a transdisciplinary communication between dentistry and metabolic diseases [194].

Current management of type 2 diabetes aims to control hyperglycemia and reduce diabetes symptoms. However, antidiabetic pharmaceutical compounds have undesirable side effects. None of these compounds specifically target the key molecular players highlighted in this review and are responsible for inflammation and oxidative stress contributing to insulin signaling pathway disruption. In this regard, plant polyphenols able to exert anti-inflammatory, antioxidant, insulin-sensitizing and anti-bacterial properties are of relevant interest to counteract the deleterious impact of periodontal bacteria and improve insulin resistance. Further studies will be needed to understand the precise impact of polyphenols in tissues targeted by bacteria in animal models and to assess their benefits in clinical trials.

## Data Availability

Not applicable.
